# Association between the frequency of different modes of delivery and depression: a national cross-sectional study

**DOI:** 10.3389/fpsyt.2025.1595829

**Published:** 2025-05-23

**Authors:** Sijie He, Houhong Li, Fan Xie, Wei Li, Li Wan, Huan Zhang

**Affiliations:** ^1^ Department of Pharmacy, Maternal and Child Health Hospital of Hubei Province, Tongji Medical College, Huazhong University of Science and Technology, Wuhan, China; ^2^ Department of Obstetrics and Gynecology, Maternal and Child Health Hospital of Hubei Province, Tongji Medical College, Huazhong University of Science and Technology, Wuhan, China

**Keywords:** depression, PHQ-9, parity, vaginal delivery, cesarean delivery, national health and nutrition examination survey

## Abstract

**Background:**

Depression is a significant mental health concern among women. The objective of this study was to explore the relationship between reproductive factors such as parity and the frequency of different modes of delivery and depression.

**Methods:**

The analysis was conducted based on the National Health and Nutrition Examination Survey (NHANES) 2005-2014, involving 5,401 non-pregnant women aged 20 years or older. Depression was evaluated using the Patient Health Questionnaire-9 (PHQ-9), while information on parity and delivery modes was self-reported. Multivariable logistic regression models were employed to investigate the association between parity, the frequency of vaginal and cesarean deliveries, and depression. Additionally, smooth curve fitting and subgroup analysis were performed.

**Results:**

After adjusting for all covariates, higher parity (OR: 1.12, 95% CI: 1.06-1.19) and an increased frequency of vaginal deliveries (OR: 1.12, 95% CI: 1.06-1.18) were both associated with a higher prevalence of depression. Women with four or more total births exhibited a 1.78-fold greater prevalence of depression relative to those with no births. Similarly, compared to women with no vaginal deliveries or cesarean sections, the prevalence was 1.81 times higher in those with four or more vaginal deliveries and 2.03 times higher in those with four or more cesarean deliveries.

**Conclusions:**

Greater parity, particularly a higher frequency vaginal deliveries, is significantly associated to an elevated prevalence of depression among women. The findings highlight the need to consider reproductive history in mental screening for women, especially those with multiple vaginal deliveries.

## 1 Introduction

Depression is a mood disorder characterized by persistent sadness and diminished capacity for pleasure accompanied by impairments in daily activities (1). Epidemiological studies consistently demonstrate a gender disparity, with women exhibiting higher prevalance compared to men (2). On the basis of this broad definition, the lifetime incidence of depression in the United States is more than 12% in men and 20% in women (3). The overall 12-month prevalence of depression is approximately 6%, while lifetime prevalence ranges from 15% to 18%, indicating that roughly 1 in 5 individuals will experience depression at some point in their lifetime (4). In the United States, 9% of adults experience major depressive disorder annually, with lifetime prevalence rates of 30% for women and 17% for men (5). Major depressive disorder places substantial economic burdens on both individual and societal levels, primarily due to high healthcare costs and lost productivity (6). The World Health Organization projects that by 2030, major depressive disorder could become the primary contributor to the global disease burden (7, 8). Depression can severely disrupt an individual’s work, education, and daily life, and may lead to serious outcomes such as suicide (9). Since depression often presents with nonspecific symptoms in its early stages, early diagnosis can be challenging. Understanding the risk factors contributing to its development is crucial for timely intervention.

The mode of delivery has been significantly linked to depression scores (10). An observational cohort study reported that depression prevalence at six weeks postpartum was higher in women who had cesarean section compared to those who had vaginal deliveries (3.79% vs. 2.35%) (11). However, no research has specifically investigated the link between the frequency of vaginal and cesarean deliveries and depression. Much of the current research on pregnancy and parity has focused on their effects on conditions such as retinopathy and nephropathy (12), periodontal disease (13), bone mineral density (14), type 2 diabetes mellitus (15, 16), and cancers (17, 18), with limited studies examining their relationship with depression. Systematic reviews have identified higher parity as a risk factor for postnatal depression (19). An observational study found a strong correlation between having four or more births and antenatal depression (β = 1.808, *P* = 0.020), but no significant link with postpartum depression (20). A previous study has explored the relationship between parity and cognitive function, depression, and chronic diseases among Chinese women with a history of childbirth, using the 15-item Geriatric Depression Scale to evaluate depressive symptoms. The study found no significant connection between parity and depression after accounting for confounding variables (21). Therefore, the correlation between parity and depression remains inconclusive.

To fill this gap, a large-scale cross-sectional study was conducted using data from the 2005–2014 National Health and Nutrition Examination Survey (NHANES) to investigate the association between parity, the frequency of vaginal and cesarean deliveries, and depression in U.S. women.

## 2 Methods

### 2.1 Data source and study population

The NHANES represents a nationally representative cross-sectional survey carried out in 2-year cycles. It comprises non-institutionalized civilian U.S. citizens who complete a comprehensive interview and undergo a medical and physiological examination, including laboratory tests. The study received approval from the National Centre for Health Statistics ethics review board, and all participants gave informed consent. All methods were performed in accordance with the relevant guidelines and regulations.

**Figure 1:**
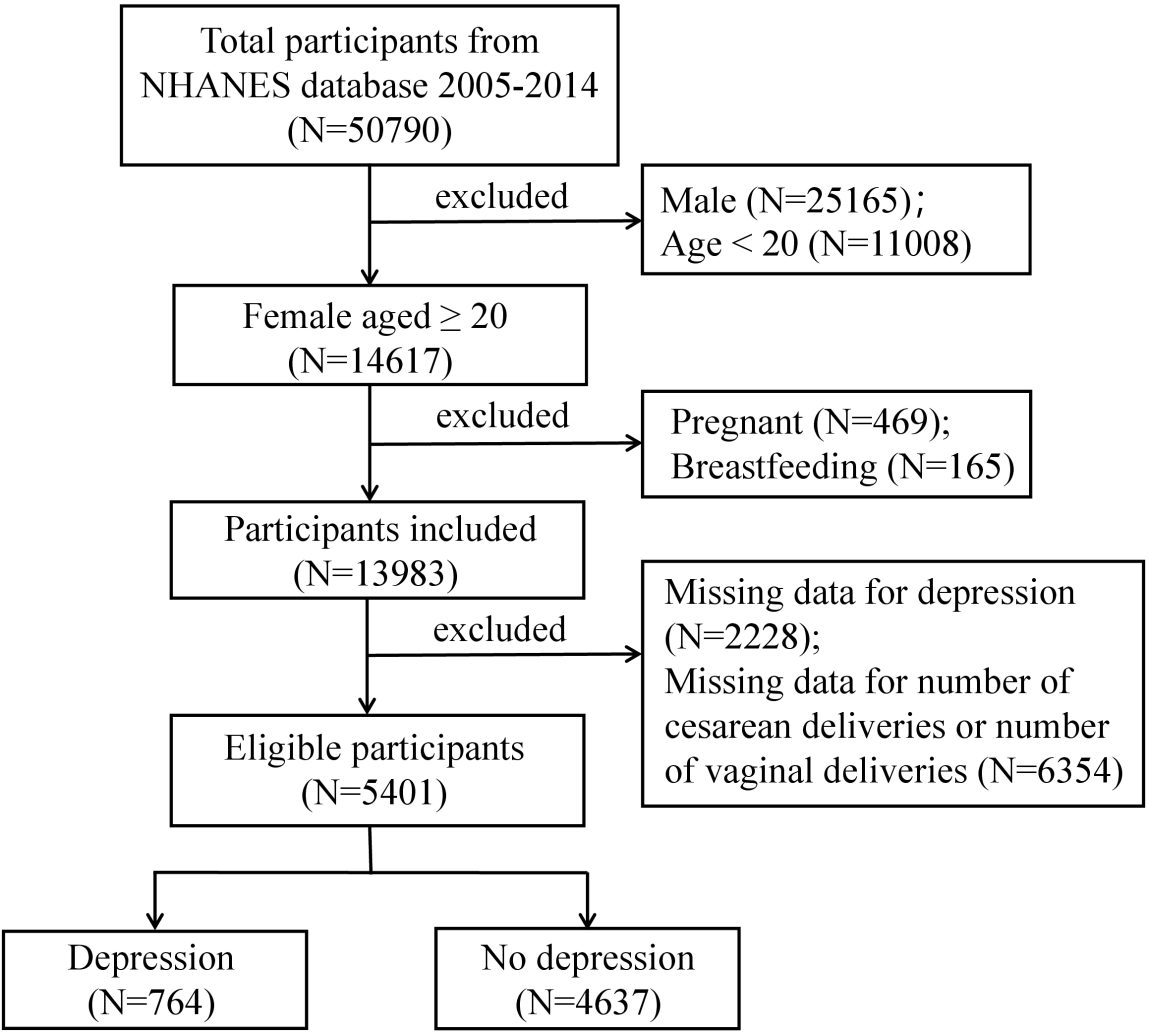
Flowchart of participants selection. A total of 50,790 participants were included, and after exclusions, the final number of participan was 5,401.

For this research, we utilized data from the 2005–2014 cycles of the NHANES, as only these cycles included detailed information on both the frequency of vaginal deliveries and cesarean deliveries. As shown in **Figure 1**, of the 50,790 individuals initially enrolled, we excluded males (n = 25,165), participants under 20 years old (n = 11,008), those who were pregnant (n = 469) or breastfeeding (n = 165), and those with incomplete data on depression (n = 2,228) or parity (n = 6,354), resulting in a final sample of 5,401 individuals.

### 2.2 Study variables

The NHANES utilizes the Patient Health Questionnaire (PHQ-9) to assess the presence and severity of depression among participants. This self-reported questionnaire evaluates the past two weeks using nine criteria from the DSM-IV for diagnosing depression. Each of the nine questions is rated on a scale from “0” (not at all) to “3” (nearly every day). The total score, ranging between 0 and 27, is calculated by summing the responses to the nine questions (22). A score of 10 or above indicates the presence of depression, while a score below 10 suggests its absence (23, 24). The frequency of vaginal deliveries is assessed using the questionnaire item coded RHQ166 (“How many vaginal deliveries have you had?”), and the frequency of cesarean deliveries is captured through RHQ169 (“How many cesarean deliveries have you had?”). Both measures include stillbirths and live births. The sum of these two values represents the total parity. Based on previous research and clinical practice experience, several potential confounding factors that could influence the association between parity, the frequency of vaginal deliveries and cesarean deliveries, and depression were accounted for (25), including demographic characteristics, lifestyle factors, physical examinations, and physical conditions. Demographic factors considered in this study included age, race/ethnicity, marital status, and education level. Race/ethnicity was divided into four categories: Mexican American, non-Hispanic White, non-Hispanic Black, and other races. Marital status was categorized as married/living with partner, widowed/divorced/separated, and never married. Education level was grouped into two categories: less than high school and high school or higher. Lifestyle factors included smoking status, which was defined as either “no” (smoked fewer than 100 cigarettes in life) or “yes” (smoked at least 100 cigarettes in life). Physical examination measures focused on body mass index (BMI). Physical conditions included diabetes, hypertension, and cancer or malignancy.

### 2.3 Statistical analysis

All estimates were calculated using sampling weights following the National Center for Health Statistics guidelines, ensuring the representativeness of the NHANES data for the civilian non-institutionalized populations in the U.S. These weights were adjusted by dividing by the number of cycles included. The mean ± SD was used for continuous variables to express central tendency and dispersion. Group differences in baseline data were assessed using t-tests for continuous variables with a normally distributed and the Mann-Whitney U test for non-normally distributed variables in two-group comparisons. For multi-group comparisons, One-way ANOVA was used for continuous variables with a normal distribution, while the Kruskal-Wallis test was applied for continuous variables with a non-normal distribution. Categorical variables were expressed as percentages and analyzed using the χ^2^ test. Weighted multivariable logistic regression analysis was conducted to evaluate the relationship between parity, the frequency of vaginal deliveries and cesarean deliveries, and depression. Odds ratios (ORs) and 95% confidence intervals (CIs) were computed to assess the strength and precision (26). Three models were developed for the multivariable test. Model 1 did not include any adjustments. Model 2 included adjustments for age and race/ethnicity, while Model 3 incorporated adjustments for all covariates. Smooth curve fitting was applied further to clarify the trend between the relationship between parity, the frequency of vaginal deliveries, and depression. Subgroup analyses were also performed to investigate potential differences in the relationship between parity, vaginal deliveries, and depression across different populations. Packages R (The R Foundation: http://www.r-project.org; version 4.4.1) and Empower Stats (www.empowerstats.com, X&Y solutions, Inc. Boston, Massachusetts) were applied to statistical analysis, with a *P*-less than 0.05 regarded as statistically significant.

## 3 Results

### 3.1 Baseline characteristics of the depression group versus the non-depression group

**Table 1** presents the weighted baseline characteristics of the study participants based on their depression status. Unweighted baseline data is provided in the **Supplementary Table S1** online. This study included 5,401 women aged over 20 years, with 764 being categorized as having depression and 4,637 not having it. The average age of the participants was 48.40 ± 14.81 years. Women with depression tended to be younger (46.27 years vs 48.68 years), had lower education levels (less than High school: 27.24% vs 15.64%), had lower rates of being unmarried (never married: 49.87% vs 64.82%) and higher rates of obesity (≥ 30: 51.69% vs 39.32%). Additionally, they showed a higher prevalence of diabetes (16.20% vs 8.04%), hypertension (45.20% vs 32.00%), and cancer or malignancy (14.08% vs 10.88%), and were more likely to have a smoking history (61.78% vs 43.67%).

**Table 1:** Baseline characteristics of study participants based on the presence of depression.

Characteristic	Total	Non- depression	Depression	*P* value
Age (years)	48.40 ± 14.81	48.68 ± 14.87	46.27 ± 14.20	< 0.001
Race/Ethnicity (%)				0.002
Mexican American	7.63	7.46	8.88	
Non-Hispanic White	65.64	66.49	59.22	
Non-Hispanic Black	14.68	14.16	18.60	
Other races	12.05	11.89	13.30	
Education level (%)				< 0.001
< High school	17.00	15.64	27.24	
≥ high school	83.00	84.36	72.76	
Marital status (%)				< 0.001
Married/Living with partner	9.88	9.28	14.37	
Widowed/Divorced/Separated	27.05	25.90	35.76	
Never Married	63.07	64.82	49.87	
BMI (%)				< 0.001
< 25	31.24	32.31	23.11	
25 ≤ -30	28.01	28.37	25.20	
≥ 30	40.75	39.32	51.69	
Diabetes (%)				< 0.001
No	91.01	91.96	83.80	
Yes	8.99	8.04	16.20	
Hypertension (%)				< 0.001
No	66.45	68.00	54.80	
Yes	33.55	32.00	45.20	
Cancer or malignancy (%)				0.017
No	88.75	89.12	85.92	
Yes	11.25	10.88	14.08	
Smoked at least 100 cigarettes in life (%)				< 0.001
No	54.21	56.33	38.22	
Yes	45.79	43.67	61.78	
Parity	2.23 ± 1.55	2.20 ± 1.52	2.47 ± 1.73	< 0.001
Frequency of vaginal deliveries	1.58 ± 1.67	1.55 ± 1.65	1.80 ± 1.80	< 0.001
Frequency of cesarean deliveries	0.65 ± 0.95	0.65 ± 0.94	0.67 ± 1.04	0.600

Mean ± SD for continuous variables: *P* value was calculated by Mann-Whitney U test; % for categorical variables: *P* value was calculated by weighted χ^2^ test.

BMI, body mass index.

### 3.2 Baseline characteristics according to parity categories

The study population was divided into five parity groups: nulliparous (no births), one birth, two births, three births, and four or more births. Significant differences in baseline characteristics were observed across these groups (**Table 2**). Higher parity was associated with older age, a higher proportion of Mexican Americans, lower educational attainment, and lower marriage rates (*P* < 0.05). Additionally, women with higher parity had greater BMI and higher rates of diabetes, hypertension, cancer, smoking history, and depression (*P* < 0.05).

**Table 2:** Baseline characteristics according to parity categories.

Characteristic	Total	0	1	2	3	≥ 4	*P* value
Age (years)	48.40 ± 14.81	43.12 ± 15.18	44.65 ± 14.47	47.82 ± 13.25	49.72 ± 14.03	56.68 ± 15.65	< 0.001
Race/Ethnicity (%)							< 0.001
Mexican American	7.63	3.85	5.58	6.35	9.21	13.78	
Non-Hispanic White	65.64	71.01	63.72	69.87	64.66	55.77	
Non-Hispanic Black	14.68	15.30	16.52	12.75	13.83	17.47	
Other races	12.05	9.84	14.18	11.03	12.30	12.98	
Education level (%)							< 0.001
< High school	17.00	9.63	11.20	13.90	19.99	32.85	
≥ high school	83.00	90.37	88.80	86.10	80.01	67.15	
Marital status (%)							< 0.001
Married/Living with partner	9.88	25.38	14.591	6.27	6.41	5.24	
Widowed/Divorced/Separated	27.05	21.06	25.481	25.01	26.60	38.89	
Never Married	63.07	53.56	59.928	68.72	66.99	55.87	
BMI (%)							< 0.001
< 25	31.24	38.37	33.71	32.78	28.56	22.99	
25 ≤ -30	28.01	26.51	29.41	26.890	29.07	28.32	
≥ 30	40.75	35.12	36.88	40.32	42.37	48.69	
Diabetes (%)							< 0.001
No	91.01	95.23	93.46	92.00	91.25	82.08	
Yes	8.99	4.77	6.54	8.00	8.75	17.92	
Hypertension (%)							< 0.001
No	66.45	72.82	72.87	67.23	65.59	52.81	
Yes	33.55	27.18	27.13	32.77	34.41	47.19	
Cancer or malignancy (%)							0.003
No	88.74	90.25	89.61	89.99	87.56	85.33	
Yes	11.26	9.75	10.39	10.01	12.44	14.67	
Smoked at least 100 cigarettes in life (%)							0.001
No	54.21	48.22	51.36	55.48	57.07	55.47	
Yes	45.79	51.78	48.64	44.52	42.93	44.53	
Depression (%)							< 0.001
No	88.30	89.69	90.07	88.87	88.24	83.78	
Yes	11.70	10.31	9.93	11.13	11.76	16.22	

Mean ± SD for continuous variables: *P* value was calculated by Kruskal-Wallis test; % for categorical variables: *P* value was calculated by weighted χ^2^ test.

BMI, body mass index.

### 3.3 Linear relationship between parity and depression

**Figure 2:**
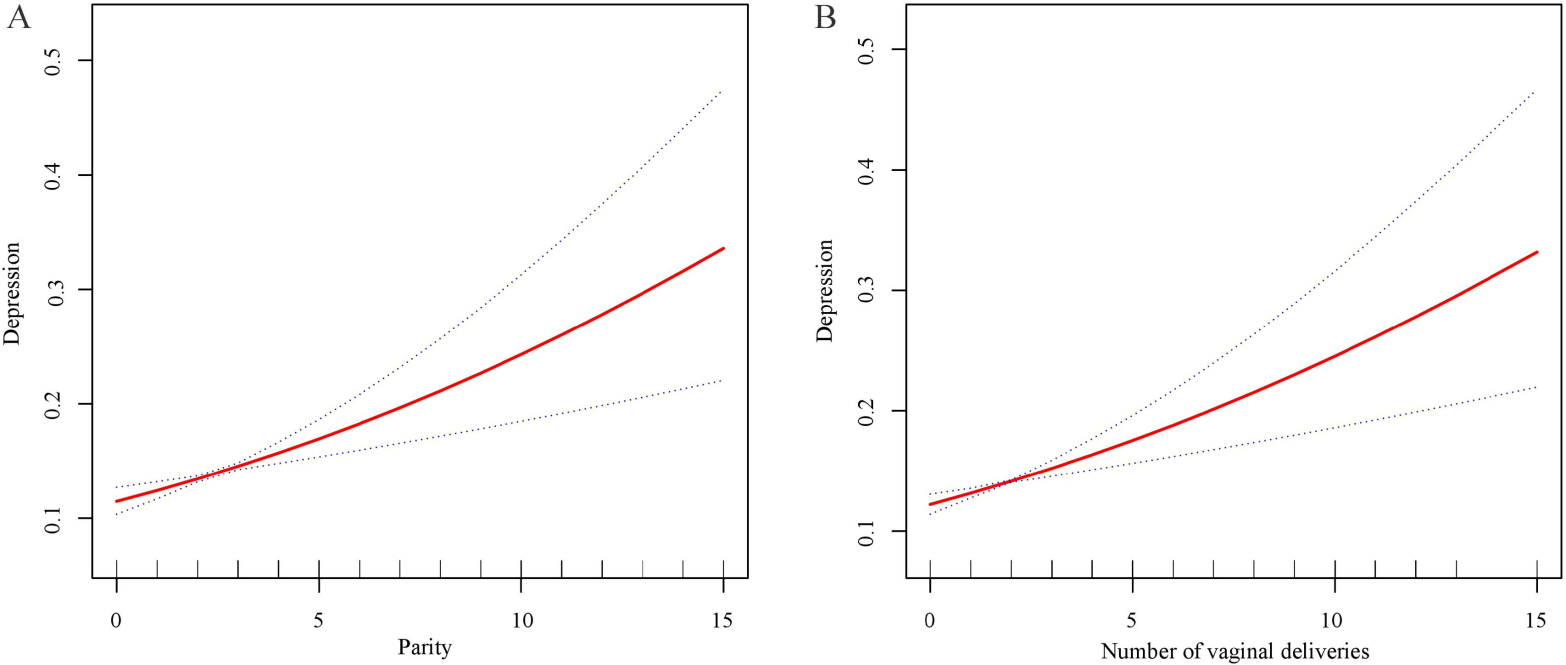
Smooth curve fittings. **(A)** The trend of the association between parity and depression. **(B)** The trend of the association between the frequency of vaginal deliveries and depression.

**Table 3** presents the ORs, 95% CIs, and corresponding *P* values of the relationship between parity and depression. In the unadjusted model, each additional birth corresponded to an 11% rise in the prevalence of depression (OR: 1.11, 95% CI: 1.06-1.17). In Model 2, which accounted for age and race/ethnicity, the prevalence of depression increased by 16% with each additional birth (OR: 1.16, 95% CI: 1.09-1.122). In Model 2, higher parity was consistently associated with a higher prevalence of depression (OR: 1.12, 95% CI: 1.06-1.19). Furthermore, in Model 3, women who had given birth four or more times experienced a 1.78-fold higher prevalence of depression than women who had never given birth. As shown in **Figure 2A**, smooth curve fittings demonstrated a strong linear relationship between parity and depression.

**Table 3:** Association between parity and depression.

Variable	Model 1		Model 2		Model 3	
	OR (95%CI)	*P* value	OR (95%CI)	*P* value	OR (95%CI)	*P* value
Parity	1.11 (1.06 1.17)	< 0.001	1.16 (1.09, 1.22)	< 0.001	1.12 (1.06, 1.19)	< 0.001
Categories						
0	Reference		Reference		Reference	
1	0.96 (0.64, 1.43)	0.839	0.97 (0.65, 1.46)	0.890	0.99 (0.65, 1.50)	0.960
2	1.09 (0.75, 1.59)	0.659	1.18 (0.80, 1.74)	0.403	1.23 (0.83, 1.84)	0.305
3	1.16 (0.78, 1.73)	0.470	1.28 (0.85, 1.93)	0.243	1.28 (0.84, 1.97)	0.257
≥ 4	1.68 (1.15, 2.46)	0.007	2.02 (1.35, 3.04)	0.001	1.78 (1.16, 2.71)	0.008
*P* for trend	< 0.001		< 0.001		0.002	

Model 1 adjusted for none. Model 2 adjusted for age and race/ethnicity. Model 3 adjusted for age, race/ethnicity, education level, marital status, BMI, diabetes, hypertension, cancer or malignancy, and smoked at least 100 cigarettes in life.

### 3.4 Association between the frequency of vaginal deliveries and cesarean deliveries with depression

The relationship between the frequency of vaginal deliveries and cesarean deliveries and depression was further explored (**Table 4**). In Model 1, each additional vaginal delivery increases the prevalence of depression by 9% (OR: 1.09, 95% CI: 1.04-1.14). After adjusting for age and race/ethnicity in Model 2, each additional vaginal delivery increases the prevalence of depression by 14% (OR: 1.14, 95% CI: 1.08-1.20). In the fully adjusted model, this positive correlation persisted, with each additional vaginal delivery increasing the prevalence of depression by 12% (OR: 1.12, 95% CI: 1.06-1.18). Specifically, the findings indicated significant positive associations among women with two (OR: 1.55, 95% CI: 1.15-2.10), three (OR: 1.46, 95% CI: 1.04-2.07), and four or more times (OR: 1.81, 95% CI: 1.31-2.51) vaginal deliveries compared to those with no vaginal deliveries. Smooth curve fittings further confirmed a linear relationship between vaginal delivery frequency and depression (**Figure 2B**). Additionally, when the frequency of cesarean deliveries was treated as a continuous variable, no significant association with depression was observed (**Table 4**). However, after converting the frequency of cesarean sections into a categorical variable, women who had undergone four or more cesarean deliveries had a 2.03 times higher depression prevalence compared to individuals who had never experienced a cesarean section (OR: 2.03, 95% CI: 1.01-4.10) (**Table 4**).

**Table 4:** Association between the frequency of vaginal deliveries, the frequency of cesarean deliveries, and depression.

Variable	Model 1		Model 2		Model 3	
	OR (95%CI)	*P* value	OR (95%CI)	*P* value	OR (95%CI)	*P* value
Frequency of vaginal deliveries	1.09 (1.04, 1.14)	< 0.001	1.14 (1.08, 1.20)	< 0.001	1.12 (1.06, 1.18)	< 0.001
Categories						
0	Reference		Reference		Reference	
1	1.28 (0.96, 1.71)	0.093	1.32 (0.99, 1.77)	0.061	1.30 (0.96, 1.77)	0.089
2	1.38 (1.04, 1.82)	0.025	1.51 (1.13, 2.01)	0.005	1.55 (1.15, 2.10)	0.004
3	1.30 (0.94, 1.79)	0.111	1.47 (1.05, 2.04)	0.024	1.46 (1.04, 2.07)	0.030
≥ 4	1.61 (1.22, 2.13)	0.001	2.00 (1.47, 2.74)	< 0.001	1.81 (1.31, 2.51)	< 0.001
*P* for trend	< 0.001		< 0.001		< 0.001	
Frequency of cesarean deliveries	1.02 (0.92, 1.14)	0.666	1.00 (0.90, 1.11)	0.957	0.96 (0.86, 1.08)	0.518
Categories						
0	Reference		Reference		Reference	
1	0.88 (0.69, 1.13)	0.315	0.83 (0.64, 1.06)	0.135	0.80 (0.62, 1.05)	0.102
2	0.88 (0.65, 1.18)	0.379	0.82 (0.61, 1.11)	0.201	0.78 (0.57, 1.07)	0.121
3	1.00 (0.63, 1.59)	0.988	0.94 (0.59, 1.51)	0.804	0.82 (0.49, 1.37)	0.444
≥ 4	2.54 (1.33, 4.85)	0.005	2.34 (1.22, 4.49)	0.010	2.03 (1.01, 4.10)	0.048
*P* for trend	0.478		0.803		0.473	

Model 1 adjusted for none. Model 2 adjusted for age and race/ethnicity. Model 3 adjusted for age, race/ethnicity, education level, marital status, BMI, diabetes, hypertension, cancer or malignancy, and smoked at least 100 cigarettes in life.

### 3.5 Subgroup analysis

The robustness of the relationship between parity, the frequency of vaginal deliveries, and depression was further evaluated by subgroup analyses. A significant association between parity and depression was identified among women aged 20–40 years (OR: 1.20, 95% CI: 1.07-1.35), those with a BMI < 25 (OR: 1.26, 95% CI: 1.13-1.41), a BMI between 25 and 30 (OR: 1.18, 95% CI: 1.05-1.33), women without hypertension (OR: 1.21 95% CI: 1.11-1.31), and those who had smoked at least 100 cigarettes in life (OR: 1.17, 95% CI: 1.08-1.26) (**Table 5**). While no associations were found in women aged 41–55 years and 56–85 years, those with a BMI ≥ 30, hypertension, or those who had a smoking history. Moreover, the relationship between parity and depression persisted consistently across the rest of the subgroups, including race/ethnicity, education level, marital status, diabetes, and cancer or malignancy (*P* for interaction > 0.05) (**Table 5**).

**Table 5:** Subgroup analysis for the associations between parity and depression.

Subgroups	OR (95%CI)	*P* value	*P* for interaction
Age			0.042
Tertile 1 (20-40)	1.20 (1.07, 1.35)	0.002	
Tertile 2 (41-55)	1.09 (0.97, 1.23)	0.159	
Tertile 3 (56-85)	1.04 (0.96, 1.12)	0.359	
Race/Ethnicity			0.297
Mexican American	1.16 (1.04, 1.29)	0.006	
Non-Hispanic White	1.11 (1.01, 1.22)	0.029	
Non-Hispanic Black	1.11 (1.00, 1.23)	0.050	
Other races	1.05 (0.91, 1.21)	0.515	
Education level			0.333
< High school	1.12 (1.04, 1.22)	0.004	
≥ high school	1.11 (1.03, 1.21)	0.011	
Marital status			0.704
Married/Living with partner	1.14 (0.95, 1.38)	0.164	
Widowed/Divorced/Separated	1.13 (1.04, 1.23)	0.003	
Never Married	1.13 (1.03, 1.24)	0.012	
BMI			0.027
< 25	1.26 (1.13, 1.41)	< 0.001	
25 ≤ -30	1.18 (1.05, 1.33)	0.006	
≥ 30	1.02 (0.94, 1.12)	0.580	
Diabetes			0.390
No	1.12 (1.05, 1.20)	0.001	
Yes	1.12 (1.00, 1.25)	0.044	
Hypertension			0.048
No	1.21 (1.11, 1.31)	< 0.001	
Yes	1.05 (0.96, 1.13)	0.286	
Cancer or malignancy			0.364
No	1.13 (1.06, 1.20)	< 0.001	
Yes	1.12 (0.94, 1.33)	0.200	
Smoked at least 100 cigarettes in life			0.005
No	1.06 (0.97, 1.16)	0.182	
Yes	1.17 (1.08, 1.26)	< 0.001	

Model 1 adjusted for none. Model 2 adjusted for age and race/ethnicity. Model 3 adjusted for age, race/ethnicity, education level, marital status, BMI, diabetes, hypertension, cancer or malignancy, and smoked at least 100 cigarettes in life.

BMI, body mass index.

Similarly, a significant relationship between vaginal delivery frequency and depression was observed among women aged 20–40 years (OR: 1.24, 95% CI: 1.11-1.38) and 42–56 years (OR: 1.12, 95% CI: 1.01-1.24), women with a BMI < 25 (OR: 1.25, 95% CI: 1.12-1.39), a BMI between 25 and 30 (OR: 1.19, 95% CI: 1.06-1.33), those without cancer or malignancy (OR: 1.14, 95% CI: 1.08-1.21), and those who had a smoking history (OR: 1.19, 95% CI: 1.11-1.28) (**Table 6**). No significant associations were found among women aged 41–55 years, 56–85 years, those with a BMI ≥ 30, cancer or malignancy, or those who smoked less than 100 cigarettes in life. Furthermore, the relationship between vaginal delivery frequency and depression persisted consistently across race/ethnicity, education level, marital status, diabetes, and hypertension (*P* for interaction > 0.05) (**Table 6**).

**Table 6:** Subgroup analysis for the associations between the frequency of vaginal deliveries and depression.

Subgroups	OR (95%CI)	*P* value	*P* for interaction
Age			0.015
Tertile 1 (20-40)	1.24 (1.11, 1.38)	< 0.001	
Tertile 2 (41-55)	1.12 (1.01, 1.24)	0.029	
Tertile 3 (56-85)	1.00 (0.92, 1.08)	0.968	
Race/Ethnicity			0.297
Mexican American	1.13 (1.03, 1.23)	0.009	
Non-Hispanic White	1.13 (1.03, 1.23)	0.009	
Non-Hispanic Black	1.07 (0.97, 1.18)	0.180	
Other races	1.12 (0.99, 1.27)	0.081	
Education level			0.174
< High school	1.09 (1.02, 1.18)	0.019	
≥ high school	1.13 (1.04, 1.21)	0.002	
Marital status			0.308
Married/Living with partner	1.09 (0.91, 1.30)	0.348	
Widowed/Divorced/Separated	1.13 (1.05, 1.22)	0.001	
Never Married	1.13 (1.03, 1.23)	0.007	
BMI			0.031
< 25	1.25 (1.12, 1.39)	< 0.001	
25 ≤ -30	1.19 (1.06, 1.33)	0.003	
≥ 30	1.03 (0.96, 1.11)	0.425	
Diabetes			0.482
No	1.13 (1.06, 1.20)	< 0.001	
Yes	1.10 (0.98, 1.24)	0.113	
Hypertension			0.063
No	1.19 (1.10, 1.29)	< 0.001	
Yes	1.05 (0.98, 1.13)	0.185	
Cancer or malignancy			0.046
No	1.14 (1.08, 1.21)	< 0.001	
Yes	1.01 (0.84, 1.22)	0.911	
Smoked at least 100 cigarettes in life			< 0.001
No	1.02 (0.94, 1.12)	0.614	
Yes	1.19 (1.11, 1.28)	< 0.001	

Model 1 adjusted for none. Model 2 adjusted for age and race/ethnicity. Model 3 adjusted for age, race/ethnicity, education level, marital status, BMI, diabetes, hypertension, cancer or malignancy, and smoked at least 100 cigarettes in life.

BMI, body mass index.

## 4 Discussion

This study presented positive linear correlations between parity, the frequency of vaginal deliveries, and depression. The prevalence of depression was 1.78, 1.81, and 2.03 times higher in women with four or more total births, vaginal deliveries, and cesarean deliveries, respectively, compared to those with no history of childbirth, vaginal delivery, or cesarean section. The findings emphasize the critical role of parity and the frequency of vaginal deliveries and cesarean deliveries in understanding the likelihood of getting depression.

As far as we know, this is the pioneering work that looks into the association between parity, as well as the frequency of vaginal and cesarean deliveries, with depression. Current epidemiological evidence is limited. Prior research has primarily explored the association between parity and other health outcomes, including cognition (27), bone mineral density (28), inflammation (29), and thyroid autoimmunity (30). While multiple studies have identified a relationship between fertility and depression in women, particularly during the perinatal period, which is a high-risk time for developing depression (31–33), the specific impact of the frequency of vaginal or cesarean deliveries on depression remains unexplored. Bennett et al. reviewed the literature and reported that the incidence of depression during various stages of pregnancy ranges from approximately 7.4% to 12.8% (34). A secondary cohort study conducted between 2015 and 2016 identified operative vaginal delivery as a risk factor for postpartum depression (PPD) (35). Chaaya et al. also reported that vaginal deliveries were related to an increased risk of PPD (36). Consistent with these findings, our study observed that higher parity and more frequent vaginal deliveries were independently linked to a greater prevalence of depression. Nonetheless, a few studies have reported contradictory findings (21, 37, 38). Tammentie et al. conducted a study using the Edinburgh Postnatal Depression Scale (EPDS) and demographic questionnaire mailed to families in Edinburgh, concluding that factors such as parity or delivery method were not correlated with depressive symptoms (37). Similarly, a cross-sectional hospital-based survey of 2305 pregnant and post-partum women (18–48 years) identified parity as a risk factor for antenatal depressive symptoms but not for postnatal depression (20). Several factors may explain the differences between these studies and our findings. One possible reason is the variation in depression assessment tools: the studies cited above used the EPDS to evaluate prenatal and postnatal depression, while our study employed the PHQ-9, which assesses a broader range of depression symptoms. Additionally, these studies focused on different populations, which introduces potential cultural, socioeconomic, and healthcare system differences that may influence the relationship between parity and depressive symptoms. These differences warrant further investigation to fully understand the differences in findings.

The positive association between parity and the frequency of vaginal deliveries and depression may be explained through several potential mechanisms. First, repeated pregnancies and vaginal deliveries can impose cumulative physical and psychological stress on women and increase the risk of depression-related chronic health issues (39–41), such as pelvic floor dysfunction, chronic pain, fatigue, and metabolic syndrome (42). In addition, having multiple children often leads to increased parenting demands, financial strain, and caregiving responsibilities, all of which are known to contribute to depression (43). Second, hormonal changes associated with pregnancy and labor can also contribute to mood regulation, increasing the risk of depression (44, 45). PPD, a well-documented concern (46, 47), may partly explain the connection between higher parity and an elevated risk of depressive symptom. Third, high parity is often associated with greater economic challenges, longer work hours, and lower savings, which may contribute to long-term depression (21). Furthermore, the relationship between the frequency of vaginal deliveries and depression may be partially explained by the physical discomfort and pain experienced during and after procedures such as episiotomies, tear repairs, or forceps application, which could be an underlying mechanism (48).

Subgroup analyses indicated that the correlation between parity, vaginal delivery frequency, and depression was more pronounced among certain demographic groups. Women with lower BMI and younger age may experience heightened reproductive pressure, hormonal changes, and caregiving demands, all of which are known contributors to depression risk (45, 49, 50). Smokers, who may already have a higher predisposition to mental health challenges (51), could be more vulnerable to the negative effects of childbirth on mental well-being. Additionally, the association between parity and depression was more pronounced in women without hypertension, while it was not significant in those with hypertension. This may be explained by the chronic burden of hypertension masking the influence of childbirth on depression. Similarly, the correlation between vaginal delivery frequency and depression was notable among women without cancer but was not significant among cancer patients. The complex physical and psychological stressors associated with cancer may reduce the effect of delivery mode on depression risk. Further investigation is essential to verify these results and explore the pathways behind these subgroup differences.

This study possesses multiple strengths. First, it utilized nationally representative NHANES data with a large sample size, allowing generalization to the broader U.S. female population. Second, we adjusted for several potential confounding factors, which strengthens the reliability of the results. Third, detailed subgroup analyses were conducted, revealing specific relationships between parity, the frequency of vaginal deliveries, and depression in certain groups, offering a foundation for future research. However, several limitations need to be noted. As a cross-sectional study, causal relationships between depression and parity or the frequency of vaginal deliveries cannot be established. Future research could benefit from using a prospective cohort design to follow women over time, examining changes in reproductive history and depression to clarify potential causal relationships. Additionally, although multiple confounders were controlled, unmeasured variables such as social support or economic stress could still influence the observed relationship between childbirth and depression. Furthermore, self-reported data on depression and childbirth could result in recall or reporting bias, potentially affecting the reliability of the results. However, NHANES employs standardized questionnaires to minimize reporting bias. Future studies could incorporate clinical diagnoses of depression to enhance the accuracy of the findings.

## 5 Conclusion

Our findings highlight a potential connection between parity, the frequency of vaginal deliveries, the frequency of cesarean deliveries, and depression in women, with particularly significant relationships observed in certain subgroups. These findings provide valuable insights for public health policymakers and clinicians, emphasizing the need for mental health screening and targeted mental health interventions for women with a history of multiple childbirths, especially those with repeated vaginal deliveries.

## Data Availability

Publicly available datasets were analyzed in this study. This data can be found here: https://www.cdc.gov/nchs/nhanes/index.htm.
