# Supplementary material for: Association between the frequency of different modes of delivery and depression: a national cross-sectional study
Source: Front Psychiatry. 2025 May 23;16:1595829. doi: 10.3389/fpsyt.2025.1595829 (PMC12141301; doi:10.3389/fpsyt.2025.1595829)
Supplement: Supplementary file 1 [file Table1.docx]

**Supplementary Table 1** Unweighted characteristics of the study population in the NHANES 2005-2014.

| **Characteristic** | **Total**  **（n=5401）** | **No depression**  **（n=4637）** | **Depression**  **（n=764）** | ***P* value** |
| --- | --- | --- | --- | --- |
| **Age (years)** | 49.61 ± 15.65 | 49.82 ± 15.81 | 48.38 ± 14.59 | 0.019 |
| **Race/Ethnicity****, n(%)** |  |  |  | 0.264 |
| Mexican American | 824 (15.26%) | 693 (14.95%) | 131 (17.15%) |  |
| Non-Hispanic White | 2215 (41.01%) | 1922 (41.45%) | 293 (38.35%) |  |
| Non-Hispanic Black | 1362 (25.22%) | 1170 (25.23%) | 192 (25.13%) |  |
| Other races | 1000 (18.52%) | 852 (18.37%) | 148 (19.37%) |  |
| **Education level, n(%)** |  |  |  | <0.001 |
| < High school | 1348 (24.97%) | 1066 (23.00%) | 282 (36.91%) |  |
| ≥ high school | 4050 (75.03%) | 3568 (77.00%) | 482 (63.09%) |  |
| **Marital status, n(%)** |  |  |  | <0.001 |
| Married/Living with partner | 675 (12.51%) | 555 (11.98%) | 120 (15.73%) |  |
| Widowed/Divorced/Separated | 1694 (31.39%) | 1387 (29.93%) | 307 (40.24%) |  |
| Never Married | 3028 (56.11%) | 2692 (58.09%) | 336 (44.04%) |  |
| **BMI, n(%)** |  |  |  | <0.001 |
| <25 | 1450 (27.13%) | 1296 (28.20%) | 154 (20.56%) |  |
| 25≤-30 | 1503 (28.12%) | 1320 (28.72%) | 183 (24.43%) |  |
| ≥30 | 2392 (44.75%) | 1980 (43.08%) | 412 (55.01%) |  |
| **Diabetes, n(%)** |  |  |  | <0.001 |
| No | 4713 (87.31%) | 4106 (88.57%) | 607 (79.66%) |  |
| Yes | 685 (12.69%) | 530 (11.43%) | 155 (20.34%) |  |
| **Hypertension, n(%)** |  |  |  | <0.001 |
| No | 3333 (61.80%) | 2939 (63.48%) | 394 (51.64%) |  |
| Yes | 2060 (38.20%) | 1691 (36.52%) | 369 (48.36%) |  |
| **Cancer or malignancy, n(%)** |  |  |  | 0.021 |
| No | 4868 (90.23%) | 4198 (90.61%) | 670 (87.93%) |  |
| Yes | 527 (9.77%) | 435 (9.39%) | 92 (12.07%) |  |
| **Smoked at least 100 cigarettes in life, n(%)** |  |  |  | <0.001 |
| No | 3090 (57.23%) | 2776 (59.89%) | 314 (41.10%) |  |
| Yes | 2309 (42.77%) | 1859 (40.11%) | 450 (58.90%) |  |
| **Parity** | 2.49 ± 1.79 | 2.45 ± 1.75 | 2.77 ± 2.02 | <0.001 |
| **Frequency of vaginal deliveries** | 1.82 ± 1.93 | 1.78 ± 1.90 | 2.06 ± 2.13 | <0.001 |
| **Frequency of cesarean deliveries** | 0.68 ± 0.99 | 0.67 ± 0.97 | 0.71 ± 1.07 | 0.412 |

Mean±SD for continuous variables: *P* value was calculated by Mann-Whitney U test; % for categorical variables: P value was calculated by weighted χ^2^ test.

NHANES, National Health and Nutrition Examination Survey; BMI, body mass index.
